# Efficient *de novo* production of bioactive cordycepin by *Aspergillus oryzae* using a food-grade expression platform

**DOI:** 10.1186/s12934-023-02261-5

**Published:** 2023-12-09

**Authors:** Sukanya Jeennor, Jutamas Anantayanon, Sarocha Panchanawaporn, Chanikul Chutrakul, Wanwipa Vongsangnak, Kobkul Laoteng

**Affiliations:** 1 Industrial Bioprocess Technology Research Team, Functional Ingredients and Food Innovation Research Group, National Center for Genetic Engineering and Biotechnology (BIOTEC), National Science and Technology Development Agency (NSTDA), Thailand Science Park, Phahonyothin Road, Khlong Nueng, Khlong Luang, Pathum Thani 12120 Thailand; 2 Department of Zoology, Faculty of Science, Kasetsart University, Bangkok, 10900 Thailand; 3 Omics Center for Agriculture, Bioresources, Food, and Health, Kasetsart University (OmiKU), Bangkok, 10900 Thailand

**Keywords:** *Aspergillus oryzae*, Carbon sources, Cordycepin, Synthetic biology, Submerged fermentation

## Abstract

**Background:**

Cordycepin (3′-deoxyadenosine) is an important bioactive compound in medical and healthcare markets. The drawbacks of commercial cordycepin production using *Cordyceps* spp. include long cultivation periods and low cordycepin yields. To overcome these limitations and meet the increasing market demand, the efficient production of cordycepin by the GRAS-status *Aspergillus oryzae* strain using a synthetic biology approach was developed in this study.

**Results:**

An engineered strain of *A. oryzae* capable of cordycepin production was successfully constructed by overexpressing two metabolic genes (*cns1* and *cns2*) involved in cordycepin biosynthesis under the control of constitutive promoters. Investigation of the flexibility of carbon utilization for cordycepin production by the engineered *A. oryzae* strain revealed that it was able to utilize C6-, C5-, and C12-sugars as carbon sources, with glucose being the best carbon source for cordycepin production. High cordycepin productivity (564.64 ± 9.59 mg/L/d) was acquired by optimizing the submerged fermentation conditions.

**Conclusions:**

This study demonstrates a powerful production platform for bioactive cordycepin production by *A. oryzae* using a synthetic biology approach. An efficient and cost-effective fermentation process for cordycepin production using an engineered strain was established, offering a powerful alternative source for further upscaling.

**Supplementary Information:**

The online version contains supplementary material available at 10.1186/s12934-023-02261-5.

## Background

Cordycepin (3′-deoxyadenosine) is a nucleoside analog compound commonly produced by certain ascomycete fungi, such as *Cordyceps militaris, Cordyceps sinensis*, and *Aspergillus nidulans* [1]. It is a bioactive compound with therapeutic potential, such as for the modulation of immune responses [2] and anti-tumor [3], anti-diabetic [4], anti-inflammatory [5], antioxidant [6], anti-photoaging [7], and anti-microbial activities [8, 9]. Due to these positive attributes, cordycepin is considered a natural medicinal compound. It is not only used for therapeutic applications but is also exploited as a bioactive ingredient in nutraceutical and cosmeceutical products [10]. The global demand for cordycepin is continually increasing, and the market is expected to grow at a compound annual growth rate of 10.4% over the forecast period (2018–2026). It is expected to increase from 473.4 million USD in 2018 to one billion USD in 2026 (https://www.globenewswire.com). Cordycepin is commercially produced by *Cordyceps* spp., which has a slow growth rate and low cordycepin content [11, 12]. Therefore, many attempts have been made to screen and develop promising strains [13–15] and optimize cultivation conditions to improve cordycepin production yield [16–18]. Chemical synthesis is a choice for the production process, but it is an unfriendly environmental and complex process, particularly in the purification step [19]. Cordycepin production using synthetic biology is a strategic approach to overcome these limitations.

Based on comparative genomic analyses of two cordycepin-producing fungi, *C. militaris* and *A. nidulans*, three conserved genes, oxidoreductase (*cns1*), phosphohydrolase (*cns2*), and ATP phosphoribosyl transferase (*cns3*), are physically linked as a gene cluster in the genomes of these fungi and display significant roles in cordycepin biosynthesis [20]. Gene deletion analysis of exotic strains of cordycepin producers (*C. militaris* and *A. nidulans*) and the study of recombinant strains by heterologous expression in *Saccharomyces cerevisiae, Yarrowia lipolytica*, and *Metarhizium robertsii* revealed that *cns1* and *cns2* are essential for cordycepin synthesis [20–22], whereas *cns3* acts as an enhancer. For heterologous cordycepin production, besides the metabolic genes, robust host strains and practical production processes (upstream and downstream processes) are required. Therefore, a host cell with a high growth rate and sufficient metabolic capacity to leverage precursor fluxes towards the biosynthetic route has been sought to develop a cost-effective cordycepin production process.

Among the heterologous hosts generally recognized as safe, *Aspergillus oryzae* is a potential workhorse system for producing a wide range of primary and secondary metabolites [23–26]. It has greater nutritional flexibility and environmental tolerance than yeast and it secretes metabolites into the culture medium to facilitate product recovery process [27]. Moreover, it belongs to the phylum Ascomycota, and is closely related to native cordycepin producers (*Cordyceps* spp. and *A. nidulans*) that can generate adenosine 3′-monophosphate (3′-AMP), an essential precursor for cordycepin production, through the purine metabolic pathway [20] (Additional file 1: Fig. S1). This study aimed to develop a system for the heterologous production of cordycepin in *A. oryzae* through synthetic biology using a food-grade expression platform. The metabolic pathway of *A. oryzae* was engineered by overexpressing the *cns1* and *cns2* genes, which are involved in cordycepin biosynthesis in *C. militaris*. Codon optimization and control of gene expression under strong constitutive promoters were implemented to enhance the transcription of heterologous genes in *A. oryzae*. To attain the information for developing the production process, the effects of chemical and physical parameters on cordycepin production by submerged fermentation (SmF) were investigated. This study describes the biotechnological production of cordycepin using the *A. oryzae* platform, which will accelerate technological development that contributes to the functional ingredient industry.

## Methods

### Strains and cultivations

*PyrG* auxotrophic (*ΔpyrG*) strain of *A. oryzae* BCC7051 was used as the recipient cell. The auxotrophic strain was maintained on Czapek Dox (CD) medium (BD Difco, NJ, USA) supplemented with 0.5% (w/v) uridine and 0.2% (w/v) uracil. Spore inoculum of *A. oryzae* strains were prepared by growing them on polished rice at 30 °C for 5–7 d and suspending them in 0.01% (v/v) Tween 80 solution. A spore suspension at a final concentration of 10^6^ spores/mL was used as the inoculum for SmF. The semi-synthetic medium (SM), consisting of 4.0% (w/v) glucose, 0.5% (w/v) yeast extract, 0.02% (w/v) NH_4_Cl, 0.24% (w/v) KH_2_PO_4_, 0.05% (w/v) MgSO_4_·7H_2_O, 0.01% (w/v) CaCl_2_·2H_2_O, 0.0015% (w/v) FeCl_3_·7H_2_O, 0.001% (w/v) MnSO4·H_2_O, and 0.008% (w/v) ZnSO_4_·7H_2_O [28], was used as a basic medium for fungal cultivation and transformant screening.

An auxotrophic strain of *S. cerevisiae* (INVSCI) was used as the host cells to construct a recombinant plasmid using DNA assembly [29]. Yeast cells were routinely grown on YPD medium consisting of 1% (w/v) Bacto yeast extract, 2% (w/v) Bacto peptone, and 2% (w/v) glucose. For transformant selection, SD medium lacking uracil (SD-uracil), consisting of 0.67% (w/v) Bacto yeast nitrogen base, 2.0% (w/v) glucose, and 30 mg/L of each amino acid (L-tryptophan, L-histidine, and L-leucine), was used. Yeast cultures were incubated at 30 °C with shaking at 200 rpm.

*Escherichia coli* strain DH5α was used for plasmid propagation. It was cultivated in Luria–Bertani medium (BD Difco) containing 100 µg/mL ampicillin at 37 °C with shaking at 200 rpm.

### Construction of the recombinant plasmids and cordycepin-producing strains of *A. oryzae*

Based on sequence data available in public databases (NCBI, http://www.ncbi.nlm.nih.gov/), two genes involved in cordycepin biosynthesis of *C. militaris*, oxidoreductase (*cns1;* accession number CCM04436) and phosphoribosylaminoimidazole-succinocarboxamide synthase (*cns2*; accession number CCM04437), were searched and subjected to codon usage optimization for heterologous gene expression in *A. oryzae* using the OptimumGene™ algorithm. The codon-optimized *cns1* and *cns2* genes were synthesized and fused to the pUC57 plasmid by GenScript (Piscataway, NJ, USA), yielding pCns1 and pCns2 plasmids, respectively.

A recombinant plasmid (pAoCordy) for heterologous expression in *A. oryzae* was constructed by DNA assembly via homologous recombination in the yeast *S. cerevisiae*. It contained an expression cassette of the *pyrG* marker gene for fungal transformant selection and two expression cassettes of *cns1* and *cns2* genes under the control of *AoPgpdA* [30] and *AnPgpdA* [31] constitutive promoters, respectively. These expression cassettes were flanked upstream and downstream by portions of the 5′- and 3′-untranslated region sequences (*pyrG*-LF and *pyrG*-RF) of the *pyrG* gene, respectively, to facilitate targeted integration into the fungal genome [32]. A schematic map of the expression cassette and the integration event of recombinant DNA into the *A. oryzae* genome based on double homologous recombination is presented in Additional file 1: Fig. S2.

Specific primer pairs were designed for constructing gene expression cassettes and verifying plasmid integration into the fungal genome by polymerase chain reaction (PCR), as listed in Additional file 2: Table S1. All DNA fragments were amplified using Platinum *Taq* Hi-fi DNA polymerase (Invitrogen, CA, USA) and specific primer pairs under optimized PCR conditions. The synthesized gene-containing plasmids (pCns1 and pCns2) and promoter-containing plasmids (pPgpdA1 and pAnPgpdA) [30] were used as templates to amplify the target genes (*cns1* and *cns2*) and constitutive promoters (*AoPgpdA*, and *AnPgpdA*), respectively. The respective PCR fragments were assembled with the pPyrG plasmid (14.8 kb), carrying the expression cassette of the *pyrG* marker and flanking homologous sequences specific to the *pyrG* locus on the *A. oryzae* genome [32], in the yeast cells. The pAoCordy plasmid was verified by PCR and restriction enzyme analysis and compared with the empty plasmid (pPyrG). DNA sequencing was performed to verify the sequence of the constructed plasmids. The expression cassettes of pAoCordy and pPyrG (control) were cut with the restriction enzyme *SgsI* and individually transformed into the *pyrG* auxotrophic strain of *A. oryzae* using the polyethylene-glycol-mediated method [25]. Fungal transformants grown on CD agar medium without nutrient supplementation at 30 °C for 5–7 d were picked. The precise DNA integration into the genome was determined by PCR analysis using specific oligonucleotide primers designed from the sequences of the constructed expression cassettes and the *pyrG* locus of the *A. oryzae* genome (T-5´ and T-3´ primer pairs), as shown in Additional file 1: Fig. S2.

The genetic and phenotypic stability of the selected transformants were verified by alternate subculturing on a non-selective medium (Potato Dextrose Agar; BD Difco) for five generations.

### Cultivation optimization for cordycepin production by SmF

A cordycepin-producing clone (AoCordy-T) with stable genetic and phenotypic traits was selected for SmF. Medium optimization was performed by cultivating the fungal cells in a 250-mL Erlenmeyer flask containing 50 mL of SM. Fermentation parameters were also studied using the one-factor-at-a-time method. The influence of two nucleoside precursors (adenine and adenosine) and two amino acids (glycine and glutamic acid) were investigated by adding 1 g/L of the individual supplements to the SM and incubated at 30 °C with shaking at 200 rpm for 2 d. The effects of culture temperature (27, 30, 33, 36, and 39 °C) and aeration rate (100, 150, 200, and 250 rpm) on cordycepin production were studied sequentially. The effect of the concentration of nucleoside supplementation on cordycepin production was also studied by varying the selected precursor at 0.5–2.0 g/L, and cultures were grown for 4 d.

Based on the optimal condition from the shake-flask study, the production of cordycepin by AoCordy-T in a 5-L stirred-tank bioreactor (BioFlo® 320, Eppendorf, Hamburg, Germany) was also investigated. The mycelial inoculum was prepared by culturing fungal cells in optimal medium for 24 h. The inoculum (10%, v/v) was then added to 3 L of optimal medium and cultured at 30 °C with aeration rate 200 rpm and a gas flow rate of 1.0 vvm. Samples of mycelial cells and fermented broth were periodically harvested to measure the cell growth (dry cell weight, DCW) and cordycepin titers.

### Determination of growth and cordycepin production of the AoCordy-T strain on different carbon sources

The effect of carbon source on cordycepin production by AoCordy-T cells was investigated using shake-flask cultivation. Under the optimal parameters for SmF, sugars, including C6 (glucose and fructose), C5 (xylose), and C12 (sucrose and maltose) sugars were varied to fine-tune the composition of the medium. Samples were harvested at different time points to measure the DCW, residual sugar and cordycepin concentrations. The kinetic parameters of fungal fermentation were calculated from time zero (*t*_0_) to the cultivation time point (*t*_X_) at which the cordycepin productivity in each experiment was maximal. Kinetic parameters of cell growth (specific growth rate, µ; biomass production rate, *Q*_X_; carbon consumption rate, *Q*_s_; specific rate of carbon consumption, q_S_; biomass yield on substrate, Y_X/S_) and cordycepin production (volumetric rates of cordycepin production, Q_P_; specific rate of cordycepin production, q_P_; product yield on substrate, Y_P/S_) of the engineered strain were determined using the equations described by Wannawilai et al. [33].

### Measurement of total phenolic content and antioxidant activity

The total phenolic content (TPC) and antioxidant activity of the fermented broth from the AoCordy-T and PyrG-T (control) cultures was measured. The Folin-Ciocalteu (FC) method [34] with some modifications, [26] was used to determine the total phenolic content in the culture broth. The diluted culture broth (approximately 0.5 mL) was mixed with the FC reagent and incubated at room temperature for 10 min. Then, 0.6 mL of 20% (w/v) sodium carbonate was added. After incubating the reaction mixture at 40 °C for 30 min, the absorbance of the sample was measured at 765 nm using a mixture of deionized water and the FC reagent as a blank. Gallic acid was used as the standard to generate a calibration curve for the concentration determination (R^2^ = 0.9953). TPC was expressed as milligrams of gallic acid equivalents per liter (mg GAE/L). Two antioxidant assays were performed to evaluate the antioxidant activity of cell-free broths of mycelial cultures. The 2,2′-azinobis(3-ethylbenzothiazoline-6-sulfonic acid) (ABTS) diammonium salt assay was performed as described by Biskup et al. [35]. An ABTS radical cation solution (7 mM ABTS and 2.45 mM potassium persulfate in deionized water) was prepared and incubated in the dark at 30 °C for 12–16 h. The solution was diluted with deionized water to an absorbance of 0.7 ± 0.02 at 734 nm prior to use. A mixture of cell-free broth and ABTS solution was incubated at room temperature for 6 min in the dark, and the absorbance was measured at 734 nm. The 1,1-diphenyl-2-picryhydrazyl (DPPH) assay was performed as described by Dan et al. [36]. The mixture of cell-free broth and DPPH solution was incubated at room temperature for 30 min in the dark, and the absorbance was measured at 517 nm. 6-Hydroxy- 2,5,7,8-tetramethylchroman-2-carboxylic acid (Trolox) was used as the standard, and a calibration curve (R^2^ = 0.9931) was constructed. ABTS^•+^ and DPPH^•^ radical inhibition was expressed as equivalents of Trolox (TEAC) per liter of fermented broth (mg TEAC/L). The percentage inhibition of the ABTS and DPPH radicals was determined as follows:

$${\%} \,\text{inhibition}=\left( {\text{Ab}{\text{s}_{\text{blank}}} - {\text{ }}\text{Ab}{\text{s}_{\text{sample}}}} \right)/\text{Ab}{\text{s}_{\text{blank}}} \times {\text{ }}100,$$

where Abs_blank_ is the absorbance of the mixture of deionized water and ABTS or DPPH solution and Abs_sample_ is the absorbance of the culture broth mixture with the ABTS or DPPH solutions.

### Analytical procedures

#### Determination of fungal biomass

Mycelial cells were harvested by filtration using Miracloth (EMD Chemicals, Darmstadt, Germany) and hot-air-dried at 60 °C until a constant weight was obtained.

#### Residual sugar measurement

Residual sugars in the fermented broth of the *A. oryzae* cultures were quantified by high-pressure liquid chromatography (HPLC; Ultimate 3000; Thermo Fisher Scientific, MA, USA) equipped with a refractive index detector and an Aminex™ HPX-87 H ion exclusion column (300 × 7.8 mm, 9-µm particle size; Bio-Rad Laboratories, CA, USA). The culture broth was diluted 10-fold and then filtered through a 0.2 μm sterile filter for HPLC analysis. Analysis was performed at 60 °C using 5 mM H_2_SO_4_ as the mobile phase at a flow rate of 0.6 mL/min for 30 min. Quantitative analysis of the residual sugars was performed using a standard curve correlating the area to known sugar concentrations (R^2^ > 0.9900).

#### Determination of purine nucleosides by HPLC-UV and LC-MS

Intracellular and extracellular cordycepin concentrations in the transformed cultures were quantified by HPLC-ultraviolet spectroscopy (HPLC-UV). In addition, the concentrations of the remaining adenine, adenosine, and deaminated product of cordycepin (3′-deoxyinosine) in the culture broth were determined during cultivation. Intracellular cordycepin was extracted from the mycelia using 50% methanol in distilled water as the extraction solvent and sonicated at 40 °C for 30 min. The culture broths were diluted 10-fold and then filtered through a 0.2 μm sterile filter for HPLC analysis. An HPLC instrument equipped with a Diode array detector and a C18 column (Acclaim™ 120; 5 μm, 4.6 mm x 150 mm, Thermo Fisher Scientific) was used for the analysis. The analysis was performed at a column temperature of 35 °C using 15% methanol in distilled water as the mobile phase at a flow rate of 0.7 mL/min for 12 min and detection was performed by measuring absorbance at 260 nm [37]. Cordycepin and other related nucleosides were identified by comparing their retention times with those of nucleoside standards (Sigma-Aldrich, St Louis, MO, USA), including cordycepin (catalog no. PHL82505), adenine (catalog no. A8626), adenosine (catalog no. A9251), and 3′-deoxyinosine (catalog no. TRC-D239760). The titer of each compound was measured based on calibration curves of standards at concentrations of 0.01 − 0.5 mg/mL (R^2^ > 0.9900).

The molecular mass of the recombinant products in the fermented broth of *A. oryzae* cultures was determined by liquid chromatography-mass spectrometry (LC-MS), performed using electrospray ionization mass spectrometry, a micrOTOF instrument (Bruker Daltonics®, Bremen, Germany), and an Agilent 1200 series HPLC instrument (Agilent Technologies, CA, USA). Mass spectra were determined in the positive mode using a micrOTOF mass spectrometer in the range of 100–1,000 Da.

#### Data analysis

All data are presented as mean values derived from three independent experiments. Statistical analysis was performed using Duncan’s multiple range test in the Statistical Package for the Social Sciences 11.5 program for Windows (IBM, NY, USA). Data were considered statistically significant at *p* < 0.01.

## Results

### Construction of the cordycepin-producing strain of *A. oryzae*

Using specific primer sets, 4.01- and 4.07-kb DNA fragments corresponding to the *cns1* and *cns2* expression cassettes, respectively, were detected in the recombinant plasmid (pAoCordy). In contrast, these were absent in the pPyrG backbone plasmid. Sequencing analysis revealed that the constructed expression cassettes had the correct sequences (data not shown).

The precise integration of the expression cassette into the fungal genome (*pyrG* locus) was achieved in 12 transformants (approximately 54.5% of the transformed clones). PCR analysis of these fungal transformants (AoCordy-T1 to T12) showed approximately 3.4- and 2.9-kb DNA fragments, corresponding to the 5′ and 3′ regions of the targeted *pyrG* homologous integration (Additional file 1: Fig. S2), indicating that they were true transformants (data not shown).

**Fig. 1:**
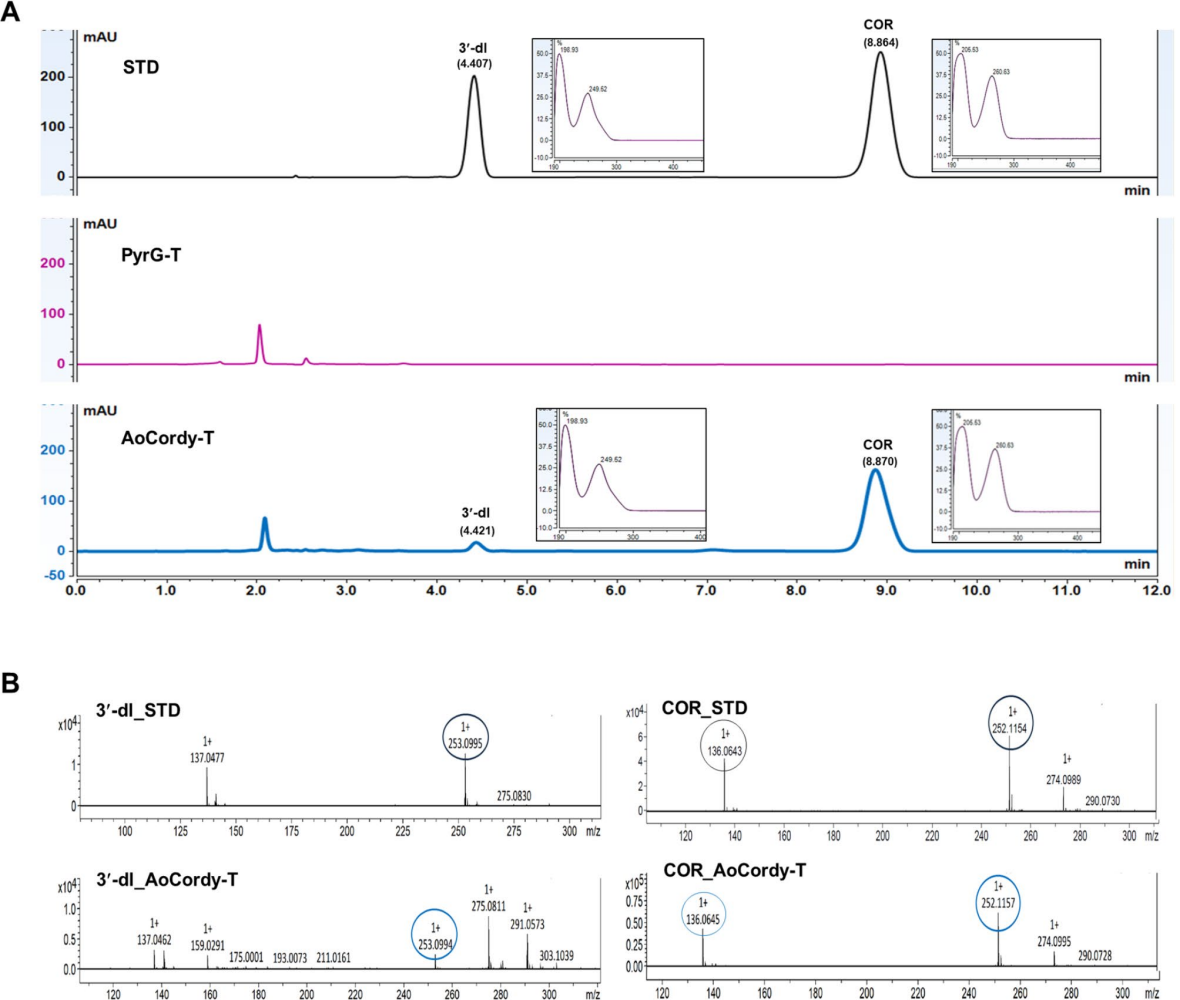
Cordycepin (COR) and 3′-deoxyinosine (3′-dl) analysis in culture broth of the engineered *A. oryzae* strain (AoCordy-T1). The chromatogram peak and UV absorption spectra (right box) of purine nucleosides analyzed by HPLC-UV are shown (**A**). The mass spectra patterns of recombinant cordycepin and its deaminated product (3′-dl) produced by the engineered strain were verified by LC-MS compared with authentic standards (STD) (**B**)

HPLC-UV analysis showed that a novel compound with a retention time of 8.87 min and absorption peaks at 206 and 260 nm, corresponding to the cordycepin standard (PHL82505, Sigma), was detected in both mycelia and cell-free broth of all AoCordy-T transformants (Fig. 1A). In addition, the deaminated product of cordycepin, 3′-deoxyinosine, with absorption peaks of 199 and 249 nm, was also found in the cell-free broth of the transformants. In contrast, these peaks were undetectable in the control clone containing the backbone plasmid (PyrG-T). LC-MS analysis confirmed that they were cordycepin and 3′-deoxyinosine, with signature molecular mass ions at *m/z* 252.1157 and 253.0994 (Fig. 1B), corresponding to the cordycepin and 3′-deoxyinosine standards (Sigma-Aldrich), respectively. As expected, these molecular mass ions were not detected in the control sample. These results clearly indicated that the engineered *A. oryzae* strain harboring heterologous *cns1* and *cns2* genes could synthesize cordycepin.

Quantification of cordycepin by HPLC-UV revealed that the engineered *A. oryzae* strain secreted cordycepin into the fermented broth rather than accumulating it in mycelial cells (intracellular cordycepin). Approximately 99.39 to 253.37 mg/L of extracellular cordycepin was detected in the fermented broths of AoCordy-T transformants, whereas the intracellular cordycepin titers were approximately 1.82 to 3.49 mg/L. The highest extracellular cordycepin production was clearly observed in the AoCordy-T1 transformant (253.37 ± 6.25 mg/L).

The genetic and phenotypic stability of the selected clone (pAoCordy-T1) was evaluated by alternate subculturing on non-selective medium (potato dextrose agar). After subculturing for five generations, 4.01-kb and 4.07-kb fragments of *cns1* and *cns2* expression cassettes, respectively, were still detected in the engineered strain (Additional file 1: Fig. S3A), indicating its genetic stability. This was supported by HPLC-UV analysis, which showed stable cordycepin production during subculturing (Additional file 1: Fig. S3B).

### Optimal SmF conditions for enhanced cordycepin production by the engineered *A. oryzae* strain

**Fig. 2:**
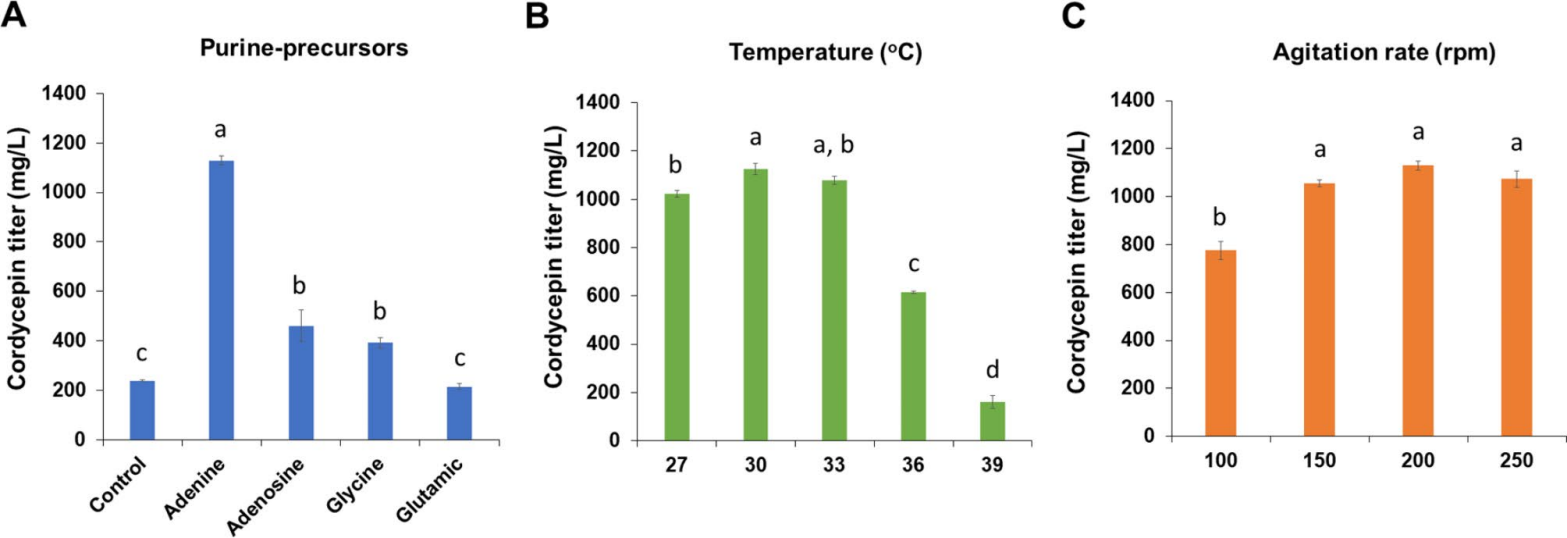
Effects of chemical and physical factors on the production of extracellular cordycepin by the AoCordy-T1 transformant. The effect of supplementation with purine precursors was investigated by individually adding 1 g/L precursor into the culture medium (**A**). The culture medium without precursor was used as a control. The effects of culture temperature (**B**) and agitation rate (**C**) were sequentially investigated using the selected adenine precursor and optimal temperature, respectively. All data are presented as mean values with standard deviation (SD). Superscript letters (a, b, c, and d) above the bars indicate statistical differences (*p* < 0.01) between the cultures with one variable factor

The effects of purine precursors and physical factors (temperature and aeration) that have been reported to trigger cordycepin production in exotic species of cordycepin producers [12] were investigated in the engineered *A. oryzae* strain. The presence of adenine, adenosine, or glycine in the SM significantly promoted (*p* < 0.01) cordycepin production by AoCordy-T1 (Fig. 2A) compared to the culture without precursor addition (control). The highest cordycepin titer (1,129.29 ± 19.17 mg/L) and productivity (564.64 ± 9.59 mg/L/d) were observed in the AoCordy-T1 culture supplemented with 1 g/L adenine, which was higher than those of the control for 4.71 folds. The effect of culture temperature on cordycepin production was investigated using medium containing 1 g/L adenine and cultivation at an agitation rate of 200 rpm. It was found that AoCordy-T1 produced a high cordycepin titer (> 900 mg/L) at a range of culture temperatures (27–33 °C). When the culture temperature reached 39 °C, the cordycepin titer of the engineered *A. oryzae* strain markedly reduced by 85.60% compared with the culture grown at 30 °C (Fig. 2B). There was no significant difference (*p* > 0.01) in cordycepin titer when cultivation was performed at agitation rates of 150–250 rpm. However, a reduction cordycepin production by approximately 31.39% was observed in the culture grown at low agitation (100 rpm) compared to the culture grown at an agitation of 200 rpm (Fig. 2C).

**Fig. 3:**
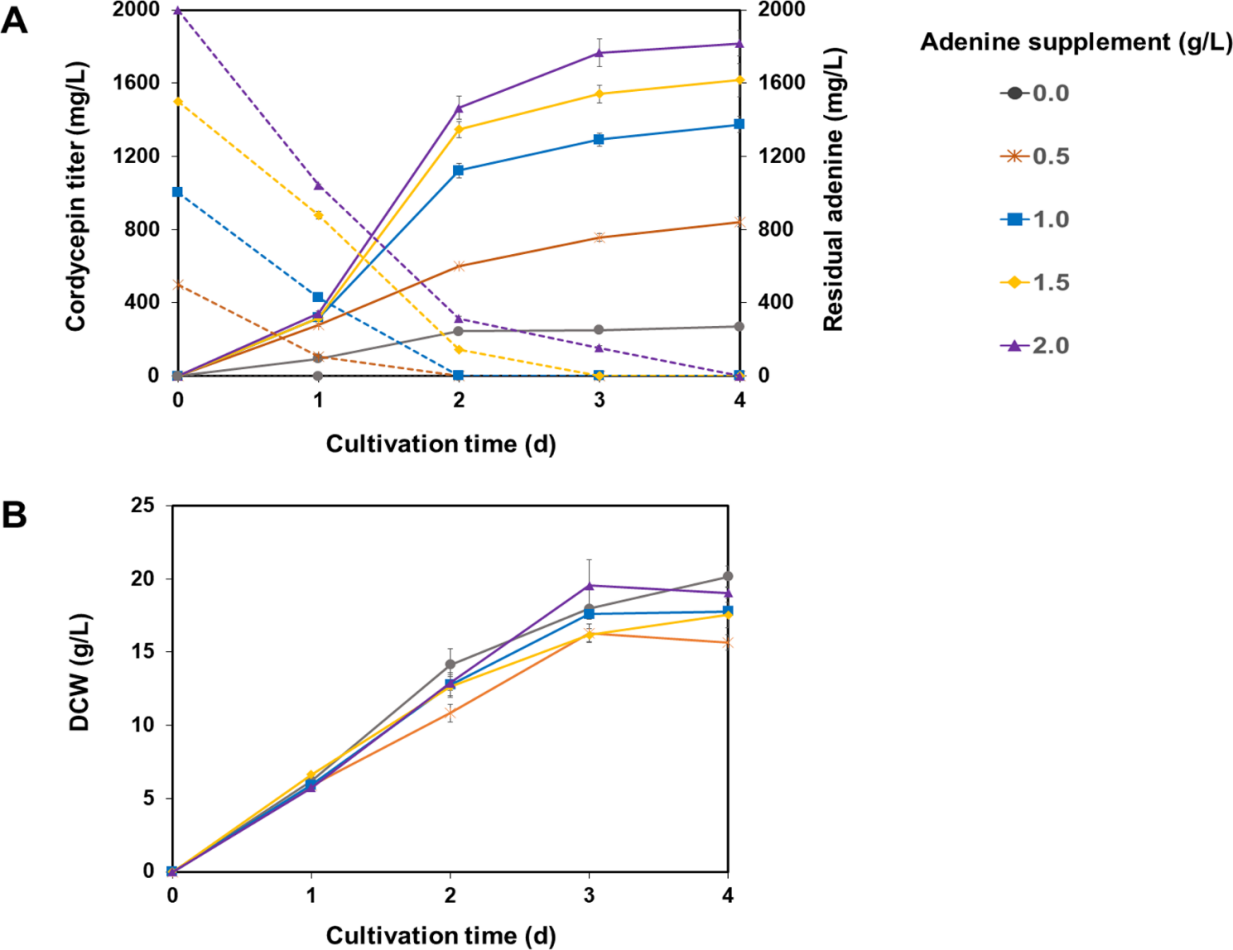
Influence of adenine concentration on cordycepin production by the AoCordy-T1 transformant. The cordycepin titer (solid line) and residual adenine (dash line) concentrations in the fermented broth (**A**) and the biomass titer (**A**) at different cultivation times are shown. The fungal cultures were grown in SM medium containing different concentrations of adenine (0.5–2.0 g/L) and were incubated at 30 °C with shaking at 200 rpm. The culture without adenine supplementation was used as a control. All data are presented as mean values with standard deviation (SD)

The influence of different adenine concentrations (0.5–2.0 g/L) on cordycepin production by the engineered strain was also investigated along with cell growth at 30 °C and 200 rpm to define the optimal cultivation time. The results showed that adenine was rapidly consumed during cell growth (Fig. 3A). The addition of different adenine concentrations did not affect mycelial growth (Fig. 3B). Cordycepin production increased with increasing adenine concentrations, with 2.0 g/L adenine showing the highest cordycepin productivity (732.31 ± 11.56 mg/L/d). An increase of 1.30- and 2.44-fold was found for cultures supplemented with 2.0 g/L adenine compared to those supplemented with 1.0 and 0.5 g/L adenine concentrations, respectively. At high levels of adenine supplementation (2.0 g/L), the remaining adenine in the culture broth was 313.34 ± 11.16 mg/L at the highest productivity point (2 d of cultivation) in contrast to the cultures supplemented with lower adenine concentrations (0.5–1.0 g/L). Therefore, we suggested adding 1.0 g/L adenine for cordycepin production regarding the supplement cost and product purification, in which 98.70% cordycepin in total nucleosides was acquired as an extracellular product of the target.

**Fig. 4:**
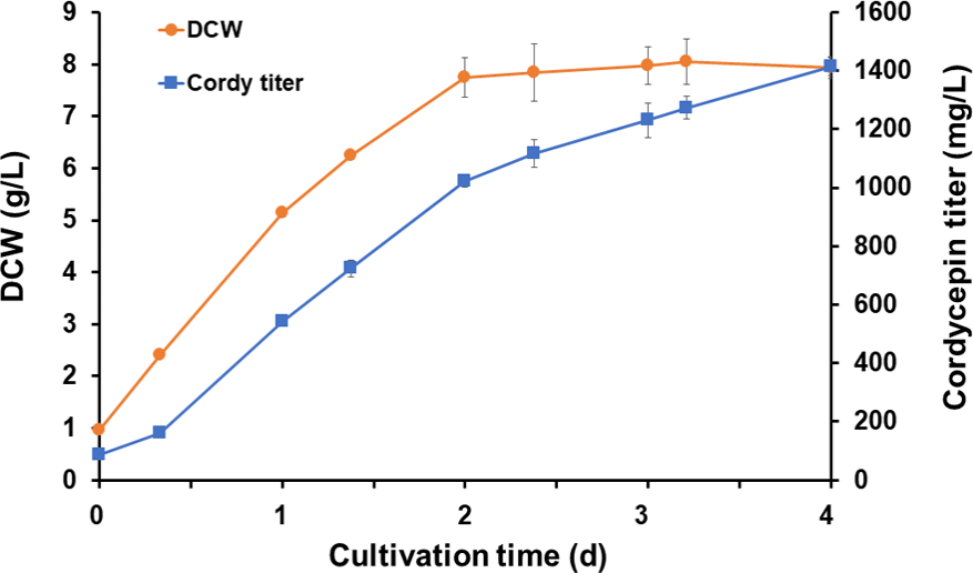
Cordycepin production and cell growth of the AoCordy-T1 transformant in a stirred-tank bioreactor. The cells were grown in SM medium supplemented with 1 g/L adenine at 30 °C with an agitation rate of 200 rpm

According to the optimal SmF conditions, the production of cordycepin by the engineered *A. oryzae* strain was investigated in a stirred-tank bioreactor using SM supplemented with 1 g/L adenine as the culture medium. Similar to the shake flask study, cordycepin production in the bioreactor markedly increased during cell growth (Fig. 4); therefore, high cordycepin productivity was observed during the 2 d cultivation (526.48 ± 19.99 mg/L/d). The highest cordycepin titer in the culture broth was found at 4 d of cultivation, reaching approximately 1,414.01 ± 30.86 mg/L. This was not significantly different from the amount produced in the shake flask experiment (1,375.00 ± 43.29 mg/L).

### Production of cordycepin by the engineered *A. oryzae* strain under different carbon sources

The utilization of various carbon sources for cordycepin production in the engineered *A. oryzae* strain was investigated by cultivation in SM supplemented with 1 g/L adenine and incubation at 30 °C with shaking at 200 rpm. The results showed that it could utilize all the carbon sources tested for cell growth and cordycepin production (Table 1). Considering the kinetic parameters of all cultivations using various carbon sources, high growth rates (*Q*_X_ and µ), cordycepin production (*Q*_P_), biomass yield (Y_X/S_), and cordycepin yield (Y_P/S_) were detected in the culture using glucose as a fermentable carbon source (Table 1). However, there was no significant difference (*p* > 0.01) in the growth performance or level of cordycepin production between the *A. oryzae* cultures using glucose, sucrose, fructose, and xylose, which exhibited maximum biomass (*C*_*X*m_) and maximum cordycepin concentrations (*C*_*Pm*_) in the range of approximately 10.85–13.64 g/L and 1,015.19–1,111.03 mg/L, respectively. A slightly lower of *C*_*Pm*_ was observed when the cells were cultured under maltose sugar (938.39 ± 3.70 mg/L). Notably, the carbon consumption rates (*Q*s, *qs*) were highest in the maltose culture, but this was not correlated with cordycepin production in the *A. oryzae* engineered strain.

**Table 1:** Kinetic parameters of the engineered *A. oryzae* strain using different carbon sources

Kinetic parameters	Carbon sources				
	Glucose	Sucrose	Fructose	Xylose	Maltose
**Volumetric productivity** (g/L d)					
*Q*_X_	6.821 ± 0.286^a^	5.945 ± 0.559^a^	5.425 ± 0.375^a^	5.730 ± 0.184^a^	5.883 ± 0.689^a^
*Q*_S_	12.199 ± 0.252^b^	12.240 ± 0.169^b^	12.345 ± 0.233^b^	12.911 ± 0.276^b^	16.647 ± 0.124^a^
*Q*_P_	0.556 ± 0.015^a^	0.524 ± 0.012^a,b^	0.508 ± 0.019^a,b^	0.530 ± 0.024^a,b^	0.469 ± 0.002^b^
**Specific rates** (g/g d)					
µ (/d)	0.971 ± 0.001^a^	0.967 ± 0.003^a^	0.964 ± 0.002^a^	0.966 ± 0.001^a^	0.967 ± 0.004^a^
*q*_S_	1.738 ± 0.035^b^	1.999 ± 0.154^b^	2.201 ± 0.188^a,b^	2.178 ± 0.021^a,b^	2.753 ± 0.292^a^
*q*_P_	0.079 ± 0.001^a^	0.086 ± 0.010^a^	0.091 ± 0.009^a^	0.089 ± 0.007^a^	0.078 ± 0.008^a^
**Yield** (g/g)					
*Y*_X/S_	0.559 ± 0.012^a^	0.485 ± 0.039^a^	0.440 ± 0.039^a,b^	0.444 ± 0.005^a,b^	0.353 ± 0.039^b^
*Y*_P/S_	0.046 ± 0.000^a^	0.043 ± 0.002^a^	0.041 ± 0.001^a^	0.041 ± 0.003^a^	0.028 ± 0.000^b^

Different superscript letters (^a, b^) above the values indicate a statistically significant difference (*p* < 0.01) in kinetic parameters between cultures using various carbon sources

The kinetic parameters were calculated from the cultures grown for 0–2 d

### Antioxidant activity of recombinant cordycepin

Based on the known antioxidant properties of cordycepin and *Cordyceps* extracts [38], the total phenolic compounds and free radical (DPPH^•^ and ABTS^•^^+^) scavenging properties of the AoCordy-T1 fermented broth were determined and compared with those of the control (PyrG-T-fermented broth). The results showed that the fermented broth of AoCordy-T1, which contained approximately 1.14 mg/mL of recombinant cordycepin, exhibited 1.19–4.57-fold higher TPC values and antioxidant activities than the control fermented broth, as illustrated in Table 2. Notably, the fermented broth of the AoCordy-T1 strain showed antioxidant properties, as it scavenged hydrophobic (DPPH) and hydrophilic (ABTS^+^) free radicals.

**Table 2:** Antioxidant properties of the fermented broth of *A. oryzae* strains

Activity	Control	AoCordy-T1	Relative fold increase*
TPC (mg GAE/L)	65.32 ± 2.23^b^	78.21 ± 1.12^a^	1.19
% Inhibition (DPPH)	54.43 ± 0.28 ^b^	65.58 ± 1.11^a^	1.20
% Inhibition (ABTS)	7.77 ± 0.67^b^	16.08 ± 1.34^a^	2.07
DPPH (mg TEAC/L)	64.80 ± 0.37^b^	79.24 ± 1.46^a^	1.24
ABTS (mg TEAC/L)	15.93 ± 4.58^b^	72.81 ± 9.16^a^	4.57

The cultures were grown in semi-synthetic medium supplemented with 1 g/L adenine and incubated at 30 °C and 200 rpm for 2 d. Different superscript letters (^a, b^) above the values indicate statistically significant differences between fungal strains (*p* < 0.01) in TPC and antioxidant activities of the fermented broths of the control and AoCordy-T1 strains

*Relative fold increase in TPC and antioxidant ability of the AoCordy-T1 fermented broth compared to those of the control (adjusted to 1.0)

## Discussion

A high-cordycepin-producing strain of *A. oryzae* for a cost-effective production process was first established through synthetic biology using informative data of the cordycepin biosynthetic pathway [20] and a food-grade expression system with antibiotic-free markers. The genetic stability of the *A. oryzae* transformant ensured the phenotypic trait of cordycepin production, even though subculturing without supplementation of the chemical signal was performed (Additional file 1: Fig. S3). Integration of cordycepin expression cassettes into the *pyrG* locus of the *A. oryzae* genome appeared to be sufficient for maintaining cordycepin production capability, in contrast to previous studies of *Aspergillus terreus* and *Xylaria* sp., which reported that the restoration of secondary metabolite production required the addition of certain chemical signals [39, 40]. Previously, constitutive promoters have been shown to be effective for awakening or enhancing the production of secondary metabolites in *A. oryzae* [41, 42]. Based on the fermentation profile of the AoCordy-T1 strain (Fig. 3), cordycepin production did not display a secondary metabolite trait, as the cordycepin titer increased with cell growth and reached a maximal level at the stationary phase. It is likely that this growth-associated metabolite depends on the promoters [30, 31] used for constitutive expression control. Cordycepin has antimicrobial activity, which affects cell survival [8, 9]. Thus, the secretion of cordycepin into the culture broth of AoCordy-T1 and the deamination of cordycepin to a non-toxic compound, 3′-deoxyinosine, by adenosine deaminase may attenuate cell toxicity [20]. A similar phenomenon was found in *C. militaris* when grown in the surface cultivation mode using a liquid medium, but not in solid-state fermentation, in which cordycepin was generated at the fruiting body stage or during nutrient starvation. It is likely that *A. oryzae* has a transport mechanism for secreting a certain amount of cordycepin into the culture broth, as more than 98% of total cordycepin was in the form of extracellular product. Genomic and phylogenetic analyses revealed that *A. oryzae* contains a large number of ABC transporters (> 70), of which the ABC-G subfamily is dominant. This subfamily of transporters has been proposed to be involved in cellular detoxification mechanisms through the exportation of natural metabolites and antifungal compounds out of the cell [20, 43, 44]. Compared to other cell factories, *A. oryzae* has the highest number of ABC-G transporters (22 transporters), whereas only 6 and 16 ABC-G transporters have been observed in *Y. lipolytica* and *A. nidulans*, respectively [43]. However, the key transporter responsible for pumping cordycepin into the culture broth has not been elucidated.

The optimal factors for mycelial cultivation using this engineered *A. oryzae* strain are essential for further evaluation of the techno-economic feasibility of cordycepin production. As cordycepin is 3′-deoxyadenosine involved in purine biosynthesis, adenine and adenosine have been proven to effectively enhance cordycepin production in various yeast and fungal strains such as *C. militaris* [18], *Y. lipolytica* [21] and *K. phaffii* [45]. We found that exogenous purine substances were also responsible for cordycepin production in the *A. oryzae* engineered strain, in which adenine addition enhanced the cordycepin production yield rather than the culture supplemented with adenosine (Fig. 2A). It is possible that adenosine was not only utilized for producing the 3′ AMP that is an intermediate for cordycepin biosynthesis, but was also used as a precursor for synthesizing other compounds through a salvage pathway [46]. In contrast, adenine was mainly converted to AMP by adenine phosphoribosyltransferase (APRT) enzyme (Additional file 1: Fig. S1). Based on transcriptome and reporter metabolite analyses in *C. militaris*, adenosine monophosphate (AMP) has been proposed as the most significant metabolite reporter (highest number of neighbors and high Z-score) related to cordycepin biosynthesis and it may be directly converted to cordycepin by omitting the 3′-AMP-associated metabolic route [47]. Therefore, cordycepin production by *A. oryzae* may prefer the AMP-associated metabolic route, as proposed in a previous study on *C. militaris* [47].

Culture temperature is an important physical factor affecting cell growth and cordycepin production [12, 48]. Although the engineered *A. oryzae* strain grew well (13.50–14.73 g DCW/L) under a temperature range (27–39 °C) similar to a previous study [26], the cordycepin titer markedly decreased in a high culture temperature (36 and 39 °C). This is likely due to the downregulation of some metabolic genes involved in cordycepin biosynthesis at such high temperatures. It has been reported that the transporters and metabolic genes associated with purine metabolism are downregulated in response to a temperature change from 28 to 41 °C in *S. cerevisiae* [49].

The engineered *A. oryzae* strain offers advantages over other cordycepin-producing strains. It had a high growth rate, rendering sufficient metabolic capacity for biotransforming the exogenous adenine precursor to cordycepin with high productivity (541.38–732.31 mg/L/d), whereas a long production period (12‒75 d) was required for cordycepin production by *C. militaris* (Table 3). Compared to yeast cell factories, *A. oryzae* showed metabolic diversity in carbon utilization (C5, C6, and C12 sugars) for cell growth (Table 1), whereas *Y. lipolytica* and *K. phaffii* have limited xylose utilization. Xylose (C5 sugar) and sucrose (C12 sugar) are renewable sugars that are considered cheap feedstock for producing value-added products. In addition, the extracellular bioproducts by filamentous fungi require only a simple unit operation for cell separation, which has an advantage over yeast cells. The cordycepin titer obtained by batch fermentation with *A. oryzae* was lower than that obtained by the recombinant yeast strains and the mutant strain of *C. militaris* (Table 3). Apart from fungal strains with different genetic backgrounds and phenotypic traits, the controllable expression system, fermentation mode, and medium composition contributed to cordycepin titer, yield, and productivity. It has been reported that methanol-inducible promoters (alcohol oxidase I; *AOX1p* and glutathione-dependent formaldehyde dehydrogenase; *FLD1p*) and defined methanol induction were used for cordycepin production by the recombinant yeast *K. phaffii* [45]. Regarding high-cordycepin-producing strain, *Y. lipolytica*, a constitutive promoter (translation elongation factor 1; *Tef1p*) was used to control cordycepin biosynthesis genes. In addition, a combinatorial approach, including enzyme fusion engineering, supply modular engineering, and fed-batch fermentation, was implemented to enhance cordycepin production [21, 50]. Enzyme fusion engineering has been proposed as an efficient strategy to improve the interaction between two enzymes and increase the production of various metabolites [51, 52]. Accordingly, the cordycepin titer of the engineered *A. oryzae* strain may be improved by the interactive function of two key enzymes: oxidoreductase and phosphoribosylaminoimidazole-succinocarboxamide synthase. Nevertheless, the high cordycepin productivity of *A. oryzae* provides a beneficial perspective regarding operating costs, particularly utility expenses. Further improvements should be directed towards bioprocess optimization (fermentation mode and downstream process) and upscaled production for techno-economic feasibility assessment to acquire crucial information for industrial practice. This study offers a practical strategy for adopting fungal systems to produce other biometabolites as functional ingredients of industrial interest.

**Table 3:** Extracellular cordycepin production by microorganisms in submerged fermentation

Organisms	Strain*	Cultivation mode	Precursor supplement (g/L)**	Cultivation time (d)	COR titer (mg/L)	COR productivity (mg/L/d)	References
A. *oryzae*	Recombinant	Batch	Adenine (1)	2	1,129.29	564.64	This study
*Y. lipolytica*	Recombinant	Fed-batch	Adenine (1)	7	4,365.54	623.23	[50]
	Recombinant	Batch	Adenosine	9	3,588.51	398.33	[21]
*K. phaffii*	Recombinant	Batch	Adenine (2)	7	2,680.00	382.86	[45]
*S. cerevisiae*	Recombinant	Batch	n/a	n/a	137.27	n/a	[22]
*E. coli*	Recombinant	Batch	n/a	n/a	nd	n/a	[20]
*C. militaris*	Wild-type	Batch	n/a	18	345.40	19.20	[53]
	Wild-type	Repeated batch	Adenine (1) +Glycine (16)	75	2,350.00	188.00	[48]
	Mutant	Repeated batch	Adenosine (6)	30	8,600.00	290.00	[18]
	Mutant	Batch	n/a	45	14,300.00	317.00	[54]
	Recombinant	Batch	Glycine (1)	12	2,581.96	215.16	[13]
*Irpex lacteus*	Wild-type	Batch	n/a	13	162.05	12.47	[55]

*Recombinant strains were constructed by overexpressing a set of genes involved in cordycepin (COR) biosynthesis. The mutant strain was constructed using proton beam irradiation

**n/a, not available; nd, not detected

## Conclusions

This study provides a perspective for the development of an efficient cordycepin-producing strain using an antibiotic-free expression system through synthetic biology, resulting in the establishment of precision fermentation for enhancing bioactive metabolite production. Using SmF, the engineered strain of *A. oryzae* with genetic stability produced a high extracellular cordycepin titer (1,129.29 ± 19.17 mg/L) and productivity (564.64 ± 9.59 mg/L/d). In addition, flexibility in carbon utilization for cordycepin production is helpful in industrial practice. Efficient cordycepin production by *A. oryzae* will accelerate the development of bioprocesses for the production of high-value cordycepin and other nucleoside-derived products with economic feasibility.

### Electronic supplementary material

Below is the link to the electronic supplementary material.

**Supplementary Material 1:** **Fig. S1:** The purine nucleotide pathway towards cordycepin biosynthesis in fungi [1, 2]. APRT, adenine phosphoribosyltransferase; AMPD, AMP deaminase; ADK, adenosine kinase; NT5E, 5’-nucleotidase; ADA, adenosine deaminase; PNP, purine nucleoside phosphorylase; PDE, phosphodiesterases; CNS1, oxidoreductase/dehydrogenase; CNS2, metal-dependent phosphohydrolase; CNS3 (ATPPRT), ATP phosphoribosyl transferase. The straight and dashed lines show validated and predicted pathways of cordycepin, respectively. Question mark indicates those reactions are unknown. **Fig. S2:** Schematic map of pAoCordy plasmid and integration event of expression cassette into A. oryzae genome based on homologous recombination mechanism. The flanking regions corresponding to the PyrG targeted locus are represented by dark-gray boxes (PyrG-LF and PyrG-RF). The dotted arrows indicate the positions of primer pairs used for determination of the integration event in engineered strain. The expected PCR fragment corresponding to the 5’- and 3’-regions of the targeted pyrG integration into the genome are shown. **Fig. S3:** Genetic and phenotypic stability of the AoCordy-T1 transformant. The spore of 1st-, 3rd- and 5th-subculturing transformants were inoculated and grown in the SM medium for 48 h. The genomic DNA of the transformant was subjected to PCR analysis for verifying its genetic stability (A). Lane M indicates a 1-kb DNA marker. Lanes 1, 3, and 5 show the amplified products of the cns1 expression cassette. Lanes 2, 4, and 6 show the amplified products of the cns2 expression cassette. The phenotypic stability in cordycepin production of the transformant was analyzed by HPLC-UV (B)

**Supplementary Material 2:** **Table S1:** Oligonucleotide primers used in this study

## Data Availability

All data generated or analyzed during this study are included in this published article and its additional files.

## List of abbreviations

3′-AMP: adenosine 3′-monophosphate
ABTS: 2,2′-azinobis(3-ethylbenzothiazoline-6-sulfonic acid)
AMP: Adenosine monophosphate
DCW: dry cell weight
DPPH: 1,1-diphenyl-2-picryhydrazyl
FC: Folin-Ciocalteu
GAE: gallic acid equivalents
HPLC: high-performance liquid chromatography
HPLC-UV: high-performance liquid chromatography-ultraviolet spectroscopy
PCR: polymerase chain reaction
SM: semi-synthetic medium
SmF: submerged fermentation
TEAC: equivalents of Trolox
TPC: total phenolic content
Trolox: 6-hydroxy- 2,5,7,8-tetramethylchroman-2-carboxylic acid
