# Supplementary material for: Efficient de novo production of bioactive cordycepin by Aspergillus oryzae using a food-grade expression platform
Source: Microb Cell Fact. 2023 Dec 9;22:253. doi: 10.1186/s12934-023-02261-5 (PMC10710699; doi:10.1186/s12934-023-02261-5)
Supplement: Supplementary file 1 — Supplementary Material 1: Fig. S1: The purine nucleotide pathway towards cordycepin biosynthesis in fungi [1, 2]. APRT, adenine phosphoribosyltransferase; AMPD, AMP deaminase; ADK, adenosine kinase; NT5E, 5’-nucleotidase; ADA, adenosine deaminase; PNP, purine nucleoside phosphorylase; PDE, phosphodiesterases; CNS1, oxidoreductase/dehydrogenase; CNS2, metal-dependent phosphohydrolase; CNS3 (ATPPRT), ATP phosphoribosyl transferase. The straight and dashed lines show validated and predicted pathways of cordycepin, respectively. Question mark indicates those reactions are unknown. Fig. S2: Schematic map of pAoCordy plasmid and integration event of expression cassette into A. oryzae genome based on homologous recombination mechanism. The flanking regions corresponding to the PyrG targeted locus are represented by dark-gray boxes (PyrG-LF and PyrG-RF). The dotted arrows indicate the positions of primer pairs used for determination of the integration event in engineered strain. The expected PCR fragment corresponding to the 5’- and 3’-regions of the targeted pyrG integration into the genome are shown. Fig. S3: Genetic and phenotypic stability of the AoCordy-T1 transformant. The spore of 1st-, 3rd- and 5th-subculturing transformants were inoculated and grown in the SM medium for 48 h. The genomic DNA of the transformant was subjected to PCR analysis for verifying its genetic stability (A). Lane M indicates a 1-kb DNA marker. Lanes 1, 3, and 5 show the amplified products of the cns1 expression cassette. Lanes 2, 4, and 6 show the amplified products of the cns2 expression cassette. The phenotypic stability in cordycepin production of the transformant was analyzed by HPLC-UV (B) [file 12934_2023_2261_MOESM1_ESM.docx]

***
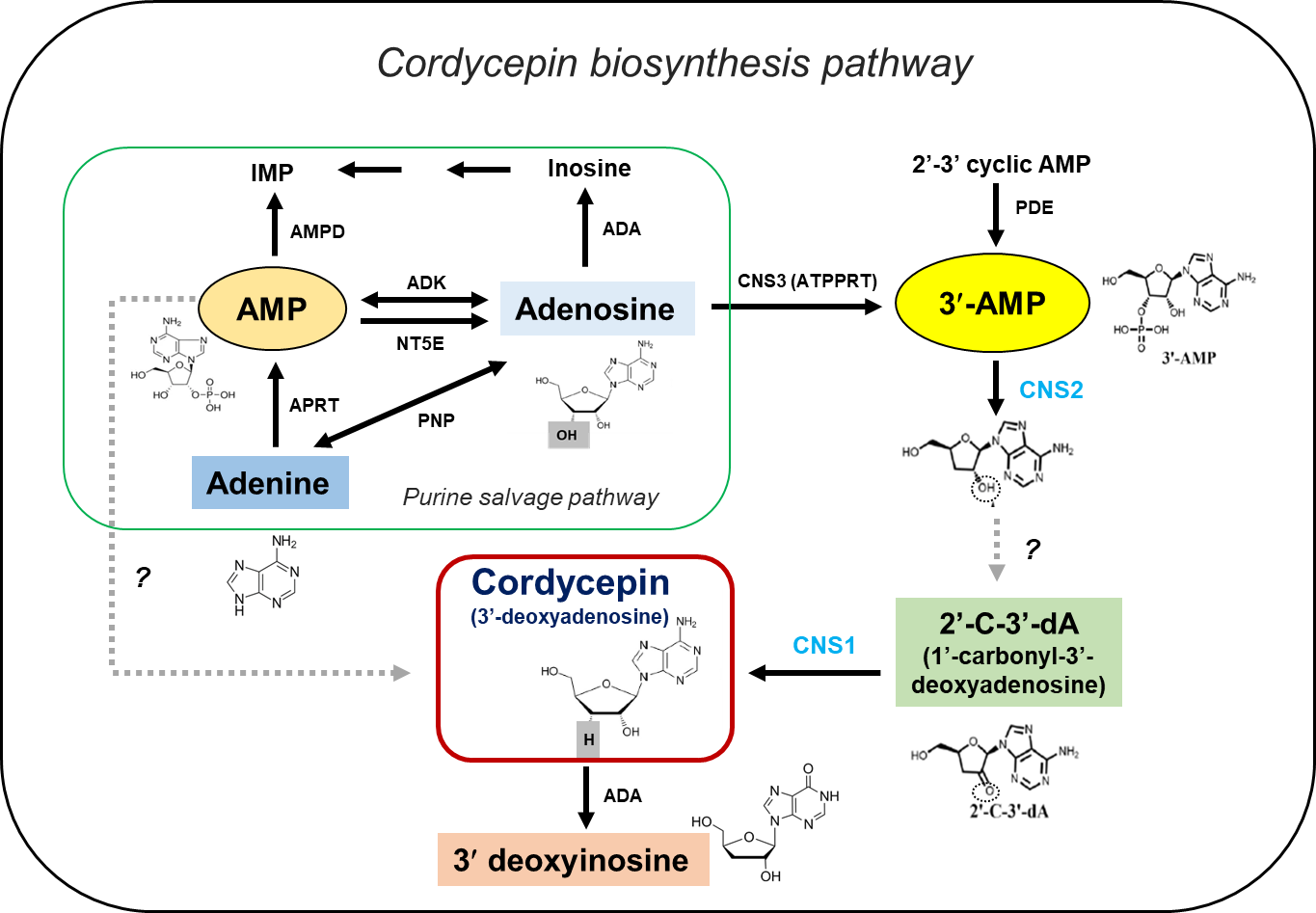
*Additional file** **1**

**Fig. S1. The purine nucleotide pathway towards cordycepin biosynthesis in fungi [1, 2].** APRT, adenine phosphoribosyltransferase; AMPD, AMP deaminase; ADK, adenosine kinase; NT5E, 5′-nucleotidase; ADA, adenosine deaminase; PNP, purine nucleoside phosphorylase; PDE, phosphodiesterases; CNS1, oxidoreductase/dehydrogenase; CNS2, metal-dependent phosphohydrolase; CNS3 (ATPPRT), ATP phosphoribosyl transferase. The straight and dashed lines show validated and predicted pathways of cordycepin, respectively. Question mark indicates those reactions are unknown.


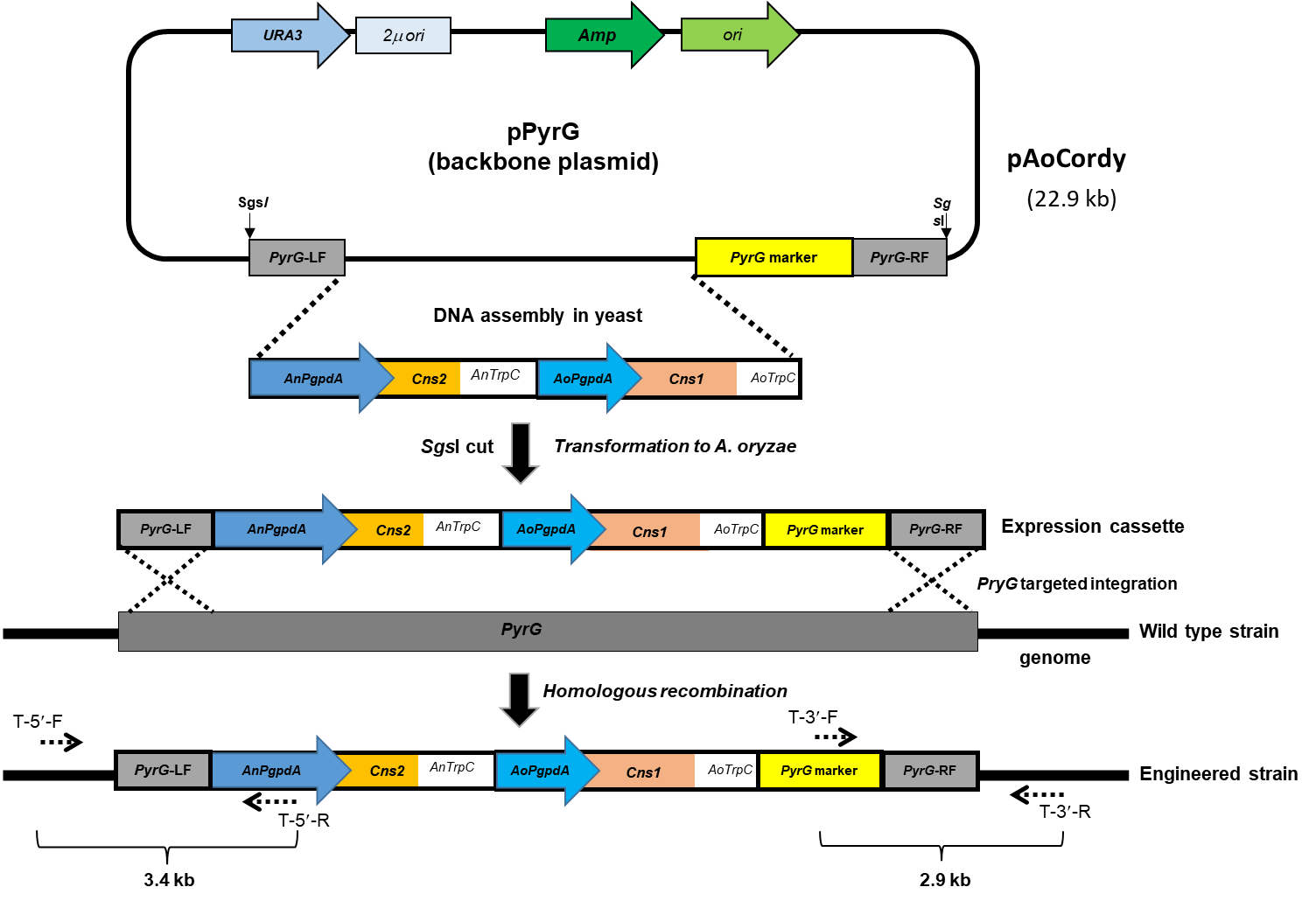


**Fig. S2.** **Schematic map of pAoCordy plasmid and integration event of expression cassette into *A. oryzae* genome based on homologous recombination mechanism.** The flanking regions corresponding to the *PyrG* targeted locus are represented by dark-gray boxes (PyrG-LF and PyrG-RF). The dotted arrows indicate the positions of primer pairs used for determination of the integration event in engineered strain. The expected PCR fragment corresponding to the 5′- and 3′-regions of the targeted *pyrG* integration into the genome are shown.


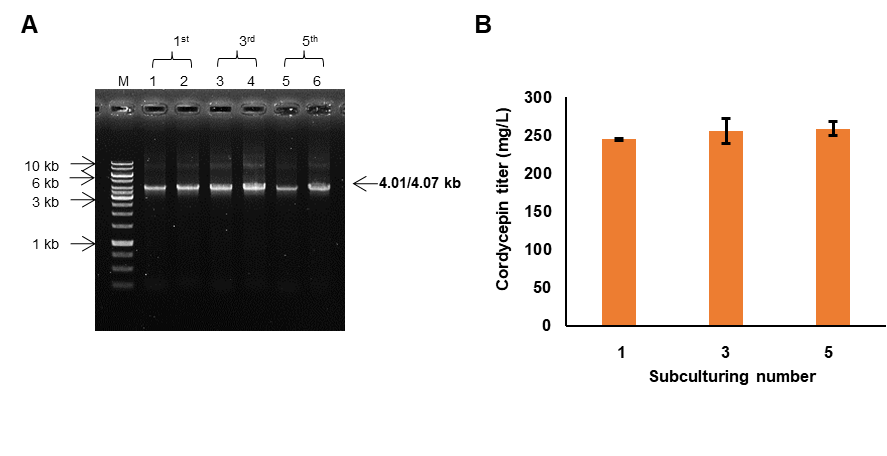


**Fig. S3. Genetic and phenotypic stability of the AoCordy-T1 transformant**. The spore of 1^st^-, 3^rd^- and 5^th^-subculturing transformants were inoculated and grown in the SM medium for 48 h. The genomic DNA of the transformant was subjected to PCR analysis for verifying its genetic stability (A). Lane M indicates a 1-kb DNA marker. Lanes 1, 3, and 5 show the amplified products of the *cns1* expression cassette. Lanes 2, 4, and 6 show the amplified products of the *cns*2 expression cassette. The phenotypic stability in cordycepin production of the transformant was analyzed by HPLC-UV (B).

**References**

1. Xia Y, Luo F, Shang Y, Chen P, Lu Y, Wang C. Fungal Cordycepin Biosynthesis Is Coupled with the Production of the Safeguard Molecule Pentostatin. Cell Chem Biol. 2017; 24:1479-1489.
2. Wongsa B, Raethong N, Chumnanpuen P, Wong-ekkabut J, Laoteng K, Vongsangnak W. Alternative metabolic routes in channeling xylose to cordycepin production of *Cordyceps militaris* identified by comparative transcriptome analysis. Genomics. 2020; 112:629–36.
