# Supplementary material for: Efficient de novo production of bioactive cordycepin by Aspergillus oryzae using a food-grade expression platform
Source: Microb Cell Fact. 2023 Dec 9;22:253. doi: 10.1186/s12934-023-02261-5 (PMC10710699; doi:10.1186/s12934-023-02261-5)
Supplement: Supplementary file 2 — Supplementary Material 2: Table S1: Oligonucleotide primers used in this study [file 12934_2023_2261_MOESM2_ESM.docx]

**Additional file** **2**

**Table S1** Oligonucleotide primers used in this study.

| **Primer Name*** | **Oligonucleotide sequence (5′−3′)**** |
| --- | --- |
| Cns1-F | caagccaaaactaccgaagaacgcaATGGCTATGAACGAGAATGCCTACC |
| Cns1-R | CTAGCTTCTTAATTGTGTCAGCAACTCAGGCGATACCCACTTTGGAGCCG |
| Cns2-F | CCGCTTGAGCAGACATCATCACCATGAGCTGCCCCACTTCGGCCGGTG |
| Cns2-R | GATGATTTCAGTAACGTTAAGTGGATCTCACCGGTGCTGGGTACGAGACAGG |
| AoPgpdA-F | GTTTAGAGGTAATCCTTCTTTCTAGAgcggccgccaatcggtccttgggagatgatc |
| AoPgpdA-R | GGTAGGCATTCTCGTTCATAGCCATTGCGTTCTTCGGTAGTTTTGGCTTG |
| AoTrpC -F | CGGCTCCAAAGTGGGTATCGCCTGAGTTGCTGACACAATTAAGAAGCTAG |
| AoTrpC -R | ggtacctagctagttagcaAAGCCTCCCAGCTGAGCATAAGTGGAC |
| AnPgpdA-F | CCTATCCTCATTTACTCCCGAGgcgatcgcCCTTGTATCTCTACACACAGGCTC |
| AnPgpdA-R | ACACCGGCCGAAGTGGGGCAGCTCATGGTGATGATGTCTGCTCAAGCGG |
| AnTrpC-F | CCTGTCTCGTACCCAGCACCGGTGAGATCCACTTAACGTTACTGAAATCATC |
| AnTrpC-R | GATCATCTCCCAAGGACCGATTGgcggccgcTCTAGAAAGAAGGATTACCTCTAAAC |
| T-5′-F | CTTGATATCCCAGAGACTGTGCATGGC |
| T-5′-R | TCTAGAAAGAAGGATTACCTCTAAAC |
| T-3′-F | TGCAGTTGTATAAGTACCTACGTACGGAC |
| T-3′-R | GTCCATGGCCAATCCCACGCAAGCAAC |

* Sense and anti-sense primers are indicated by the following F and R letters, respectively.
